# An Expanding Spectrum of Complications in Isolated Methylmalonic Aciduria

**DOI:** 10.34763/jmotherandchild.20202402si.2014.000003

**Published:** 2020-11-10

**Authors:** Patrick Forny, Stephanie Grunewald

**Affiliations:** 1 Division of Metabolism, University Children’s Hospital Zurich, Zurich, Switzerland; 2 Metabolic Medicine Department, Great Ormond Street Hospital, Institute of Child Health University College London, NIHR Biomedical Center (BRC), London, UK

**Keywords:** Methylmalonic aciduria, MMA, disease complications, renal dysfunction, neurological impairment

## Abstract

Isolated methylmalonic acidurias represent a heterogeneous genetic group of inborn errors of propionate metabolism with the common biochemical hallmark of elevated methylmalonic acid present in tissues and body fluids. It was first described in the 1960s and over the years better understanding of the disease and its presentation, earlier diagnosis, and most importantly advances in treatment have resulted in extended survival of patients. With that an expanding spectrum of complications is emerging which requires attention and regular monitoring to facilitate early intervention and reduce disease burden.

## Introduction

Isolated methylmalonic aciduria (MMA) is a group of autosomal recessive disorders caused by mutations in the genes *MMUT*, *MMAA*, *MMAB* and *MMADHC*. Depending on the causative gene and – in case of *MMUT* – residual methylmalonyl-CoA mutase (MMUT; EC 5.4.99.2) enzyme activity, the MMA subtypes are called *mut^0^*, *mut^-^*, *cblA*, *cblB*, *cblD-variant 2* (1). MMUT is the enzyme responsible for the conversion of L-methylmalonyl-CoA to succinyl-CoA in mitochondria. The vitamin B12 (cobalamin) cofactor in the form of adenosylcobalamin is required to facilitate this isomerisation reaction (2). The substrate of MMUT is derived from propionyl-CoA, which is formed by the catabolism of protein, more specifically of the amino acids isoleucine, valine, threonine and methionine, and the breakdown of the side chain of cholesterol and odd-chain fatty acids. The product of the MMUT reaction, succinyl-CoA, enters the tricarboxylic acid (TCA) cycle, also located in the mitochondria. The TCA cycle is a sequence of vital biochemical reactions serving the purpose of providing molecules, which are utilised for energy production by the respiratory chain.

The vital biochemical function of the MMUT enzyme in mitochondrial metabolism is reflected by the devastating clinical consequences it causes in case of a deficiency. Onset of the disease varies. MMA patients might present early after birth following an initial symptom-free period of a few hours to days (3). Patients may show symptoms such as poor feeding, vomiting, increasing lethargy, imitating a septic-like clinical picture, which could lead to coma and death (4). Proposed guidelines for the diagnosis and management have been published to guide physicians (5).

Methylmalonic aciduria was first recognised as an inborn error of metabolism in the 1960s (6). Raised awareness of early presenting inborn errors of metabolism, enhanced diagnostic testing and advances in clinical care over the years has improved the survival of MMA patients through the initial acute phase of the disease. In addition, there are milder MMA phenotypes which present later (late-onset, i.e. after the neonatal period), e.g. during an acute illness (7). The increased life expectancy has led to an expansion of the so far known natural history of MMA, which shows a variety of long-term complications affecting different organ systems. In addition to the well-known complications, such as neurological problems and chronic kidney disease, it is becoming more evident, that the disease course of MMA also includes some so far less anticipated complications. The occurrence of an even broader variety of complications leads to the hypothesis that almost all organ systems can be affected by MMA, depending on their susceptibility. Many of the complications can be devastating and significantly affect the quality of life of patients. Hence, this review attempts to summarise the most common as well as the emerging long-term complications of MMA disease, allowing clinicians to monitor the respective organ systems and thus aiding patient management.

## Visceral Complications

### Kidney

Renal failure is a common complication in MMA patients (3, 8). The severe subtypes (*mut^0^*, *cblB*) are more frequently affected by this complication (7, 9). Kidney function diminishes during the course of the disease as illustrated by decreasing glomerular filtration rate, which is best estimated by cystatin C measurements (10). The onset can be as early as in the second year of life (11) and needs to be monitored on a regular basis as secondary clinical complications such as renal anaemia, electrolyte disturbances, microalbuminuria and hypercalciuria might occur (12). Overall, kidney growth is also impaired (10). It was postulated, that kidney failure is based on disturbed mitophagy (13) and proximal tubule malfunction due to various OXPHOS deficiencies (14, 15). Histology showed tubulo-interstitial nephritis (16).

### Heart

As the heart is a high energy requiring organ it does not come as a surprise that this organ is affected in MMA, possibly due to impaired mitochondrial function. Although not as frequently affected, it is estimated that about 1 in 10 patients suffers from cardiomyopathy (8). The cardiomyopathy can be dilated (17, 18) or hypertrophic (8, 17). ECG might be indicative of cardiomyopathy, while QTc intervals are usually normal (8). Nevertheless, ECG abnormalities have been described including T-wave inversion and paroxysmal supraventricular tachycardia (17).

### Gastrointestinal

The most common manifestation in MMA related to the gastrointestinal (GI) tract are feeding problems which often require tube feeding or other forms of assisted feeding in up to 60% of patients (19). An often devastating and difficult to treat complication of the GI tract is pancreatitis, estimated to occur in less than 10% of MMA patients (9, 20). It often requires avoiding enteral intake for a prolonged period of time, and parenteral nutrition may be necessary (21). Pancreatitis is difficult to predict as it doesn’t seem to be related to the overall metabolic control of the patient, but occurrence of pancreatitis might be a possible predictor of mortality (20). Diagnosing pancreatitis is a challenging task as related symptoms, such as vomiting and abdominal discomfort, are frequently observed GI symptoms in MMA, particularly in unwell children. If there is any suspicion, levels of amylase and/or lipase should be checked alongside appropriate imaging modalities. The disease burden can be immense particularly when patients develop acute on chronic pancreatitis (22).

### Liver

The liver has a central role in MMA, as expression of MMUT is higher than in other organs (23). The capacity of the liver in turning over propionate metabolites is illustrated by reduction of plasma methylmalonic acid in MMA patients after liver transplantation (24). Despite the relevance of the liver in MMA, complications affecting liver function have been described only recently. Biochemical abnormalities, such as increased levels of alpha-fetoprotein and liver transaminases have been found as well as ultrasound abnormalities and signs of cirrhosis and fibrosis on liver tissue histology (25, 26). It was shown that these biochemical abnormalities can normalise when the liver is transplanted (26). Apart from these chronic and unspecific changes, five MMA patients have been published so far showing liver neoplasms, namely hepatoblastoma or hepatocellular carcinoma and in one case even a mixture of both tumour types (27, 28, 26). The neoplasms were detected at a large range of ages (4 months to 30 years) (26).

## Neurological Complications

Various aspects of neurological complications have been described in MMA. One of the most devastating complications from a neurological perspective is related to the phenomenon of metabolic stroke during which patients experience an acute onset of neurological symptoms related to stroke-like lesions in the area of the basal ganglia. It is estimated that up to one third of MMA patients experience an episode of a metabolic stroke which subsequently often results in a movement disorder (29). An acute metabolic decompensation can not only trigger a metabolic stroke, but can also lead to epilepsy. Epilepsy can manifest as part of an acute metabolic decompensation or can be triggered by brain lesions which occurred during a metabolic decompensation (9). In addition, a majority of MMA patients show impaired cognitive function, which is associated with early-onset disease and the presence of hyperammonaemia at diagnosis as well as epilepsy (30, 31, 32). Also sensory organs are involved and MMA patients show ophthalmological complications in the form of optic atrophy (8, 33) as well as sensorineural hearing loss (8, 9).

## Haematological Complications

Cytopenia of several cell lineages (mainly erythrocytes, neutrophils and thrombocytes) is commonly observed in MMA (8). It can occur during an acute metabolic crisis or chronically. Increased susceptibility to infections in the context of neutropenia has not been demonstrated in MMA patients so far. While the exact pathomechanisms causing these complications are unclear, anaemia might at least be exacerbated if renal failure is present due to insufficient synthesis of erythropoietin.

## Complications Secondary to Treatment

### Impaired growth

MMA patients often suffer from failure to thrive and poor growth (19, 34). Similar to some other complications, the phenomenon of poor growth is associated with the more severe subtype namely patients with a *mut^0^* subtype are more often and more severely afected than *mut^-^*, *cblA* and *cblB* patients (7, 34). Poor growth in MMA is multifactorial: due to the underlying disease and secondary due to the natural protein-restricted diet, which is part of the routine therapy regime. Protein intake can be overly restricted or the relation between protein and energy intake might be unbalanced, thus inhibiting normal growth (35, 36). Further, the usage of precursor-free amino acids mixtures has been discussed controversially. Growth has been negatively correlated with the amount of precursor-free amino acid mixtures being used (37). In addition, these synthetic protein mixtures are low in valine and isoleucine content, potentially inducing specific amino acid deficiencies that are related to poor growth (34).

### Bone

Low bone density is regularly observed in MMA patients (38) and is also believed to be multifactorial, and may at least partly be caused by prolonged protein-restriction. Application of amino acid supplements might support the development of decreased mineralisation of the bone via increasing the renal acid load, which induces buffering of protons in the bone tissue and thereby activating bone resorption (9).

### Neuropathy

Severe neuropathy is not a specific complication of MMA, but has been described as a side efect of long-term metronidazole treatment. This medication is sometimes used in MMA to eradicate propionate producing gut bacteria in order to lower the load of precursors (5). Toxicity has been described in other contexts, including propionic aciduria patients (39, 40), and although it is not clear how damage occurs to peripheral nerve tissue, several mechanisms have been proposed including oxidative damage, reduced synthesis of mRNA for neuronal proteins, or vitamin B1 deficiency induced by enzymatic conversion of metronidazole into a thiamine analogue (41, 42).

## Conclusions

As this review illustrates, a variety of organ systems are affected by MMA disease. Some are not discussed in detail here, such as hypermobility of the joints or muscular hypotonia, further demonstrating the ubiquitous efects of MMUT deficiency on many tissues. The exact biological mechanisms underlying tissue damage and/or malfunction are yet to be determined (9). This lack of knowledge poses problems in the development of novel therapies, which are urgently required. Available disease models (15, 43, 44) can be used to further unravel yet unknown possible disease manifestations and their pathomechanisms and to assess the efficacy of novel treatment modalities.

In the meantime, this review suggests monitoring the full range of potential long-term complications, as only their early detection allows timely symptomatic treatment.
